# Wood Plastic Composite Based on Recycled High-Density Polyethylene and Wood Waste (Sawdust)

**DOI:** 10.3390/polym16223136

**Published:** 2024-11-11

**Authors:** Camilo Oliveros-Gaviria, Edwin Cumbalaza, Jose Herminsul Mina-Hernandez, Mayra Eliana Valencia-Zapata, Juan Nicolas Suarez-Bonilla, Nicolas Martinez-Mera

**Affiliations:** 1 Escuela de Ingeniería de Materiales, Grupo Materiales Compuestos, Universidad del Valle, Calle 13 No. 100-00, Cali 760001, Colombia; camilo.oliveros@correounivalle.edu.co (C.O.-G.); edwin.cumbalaza@correounivalle.edu.co (E.C.); jose.mina@correounivalle.edu.co (J.H.M.-H.); 2 Escuela de Ingeniería Metalúrgica y Ciencia de los Materiales, Universidad Industrial de Santander, Carrera 27 Calle 9, Bucaramanga 680002, Colombia; mevalzap@uis.edu.co; 3 Diseclar: Diseño y Fabricación Ecológica, Carrera 76 No. 6-217, Cali 760001, Colombia; juan.suarez@diseclar.com

**Keywords:** wood plastic composite, high-density polyethylene, deforestation, lignocellulosic materials, coupling agents, weather resistance

## Abstract

The current work presents the reformulation of a composite based on high-density polyethylene obtained through the recycling of blow-molded containers (rHDPE) with natural fiber residues (wood sawdust). This material is technically and industrially known as WPC (wood plastic composite). The original formulation of this material contains 34% high-density polyethylene and 60% sawdust by weight fraction, while the remaining components include additives and coupling agents such as wax (Coupling Agent TPW 813 for plastic woods), stearic acid, and color pigment. The composite material was processed using the profile extrusion method, from which samples were obtained to conduct various experimental tests. The mechanical analysis revealed that both the strength and Young’s modulus of the tensile and flexural properties slightly increased with the addition of sawdust to the composite. Additionally, the stiffness was higher compared to high-density polyethylene, indicating a direct relationship between these properties and the amount of sawdust incorporated. Besides this, other characterization methods were performed on the material, including density, hardness, and compression tests, as well as differential scanning calorimetry (DSC), thermogravimetric analysis (TGA), natural and accelerated aging tests, Vicat softening temperature, and heat deflection temperature analysis (HDT). The initial evaluation provides a guide to enhance the most important properties with the aim of using the extruded profiles as pergolas in the real estate sector. Therefore, new formulations are developed with the assistance of Minitab 21 software, maintaining a constant proportion of materials that do not affect the mechanical properties, such as wax, stearic acid, and color pigment. Once the formulations are made, each one is characterized through tensile tests to determine which has the best performance. The formulation with the highest strength is re-characterized using the techniques mentioned in the starting material to obtain a material with the most optimal characteristics.

## 1. Introduction

The growing development of new materials in recent decades has gradually and systematically allowed for the replacement of other conventional materials that have accompanied humans for centuries. Wood plastic composites (WPC) are an especially interesting option for replacing conventional wood in a considerable number of applications. The consideration of seeking alternatives to the use of conventional wood is based on the fact that deforestation continues to be, in various parts of the world, an excessive and controversial activity; during the period 2010–2020, South America had an annual net loss of forests of 2.6 million hectares of forests [1]. Despite the deforestation rate in most Latin American countries having decreased compared to the last century, it remains an activity that is partly conducted illegally and without supervision or risk mitigation. These environmental problems bring with them social and economic issues as well.

On the other hand, since the beginning of the synthetic polymer science revolution in the early 20th century and its development throughout this period, there has been an immediate effect on the planet. According to a recent study by the Organization for Economic Co-operation and Development (OECD), 22% of plastic waste worldwide is mismanaged, and only 9% is successfully recycled. According to the OECD, the global production of these wastes has doubled compared to two decades ago. This translates to about 353 million tons of plastic waste generated between 2000 and 2019, representing 3.4% of total global greenhouse gas emissions [2].

Waste from the wood sawing process, such as sawdust and wood chip, also contributes to pollution. These wastes represent 0.00028% of total world trade [3]. Therefore, it is important to continue encouraging the utilization of this wood waste to avoid its environmental impacts.

The creation of a composite material based on the two types of waste presented above allows for the formation of a material from wood, natural fibers, or plastic (WPC). Its main benefit is the use of plastic waste like high-density polyethylene and lignocellulosic particles. However, previous studies show that the weather durability of wood (or natural fiber) and plastic composites (WPC) is a significant issue for outdoor applications, reflected in discoloration and loss of mechanical properties [4,5].

Current strategies to improve the properties of WPC under weathering have advanced in two directions: the first consists of additions such as nanosilica [6], coupling agents [7,8,9,10,11,12], ionic liquid additives such as bis (2,4,4-trimethylpentyl) phosphate [13], and UV stabilizers like Tinuvin 783 [14], which have shown improved UV aging resistance (photo-oxidation) due to good compatibility between components [15].

The second front that has been employed involves the implementation of processing methods such as coating, lamination, or co-extrusion to prevent the surface from being affected by the environment. It is proven that the use of these protective methods can generate environmental problems due to the use of solvents and that surface lamination on a substrate is conducted through melting or an adhesive layer, but often delamination occurs with the composite. WPCs manufactured by co-extrusion technology can have significantly improved UV-aging properties [6,15].

The strategy for improving the properties of a current WPC composite used in the local market (mention and disclose if necessary) consists in this research of the reformulation through an experimental design where the best proportions of each component necessary for the production of plastic wood were found by taking into account the use of coupling agents, which is also a known strategy to improve the mechanical properties and weather resistance of this type of composite material (as mentioned above).

## 2. Materials and Methods

### 2.1. Materials

The materials used throughout the entire research stage are those necessary for the processing of wood plastic, which are detailed in Table 1.

### 2.2. Experimental Methodology

The characterization of the current formulation was conducted through a set of tests classified into three types of analyses (physicochemical, thermal, and mechanical). Test specimens were machined from a profile supplied by Diseclar using a CNC (Computer Numerical Control) machine, with dimensions conforming to ASTM standards. A summary of the types of analysis and testing performed can be found in Table 2, Table 3 and Table 4.

Following the initial stage of characterization of the current formulation, in order to assess the effect of wood plastic composite formulation components on mechanical properties and determine the optimal blend of components, a second-order extreme vertices mixture design was implemented using Minitab 21 software (Table 5). In this mixture design, the limits were set in accordance with previous research; the lower and upper limits are identical for invariant components within each formulation:

This design resulted in 13 runs where the amount of wax, stearic acid, and coloring pigment remained constant, as shown in Table 6.

After conducting the experimental design, the weights of each component were determined for each reformulation (Table 7), taking into account the following considerations:

- The values obtained from the experimental run correspond to the weight fractions of each component.
- The effective fill factor of the available rheometer corresponds to approximately 0.72% of the total volume.
- The total volume corresponds to 69 cm^3^.

In the previous table, the weight of each component and the total sum of 25 g were shown; the same table was prepared for each of the remaining 12 blends. The weights of each component were measured on a precision balance at SENA facilities. Subsequently, the invariant components in the formulation, such as stearic acid, wax, and brown pigment, were placed in a zip-lock bag.

**Figure 1:**
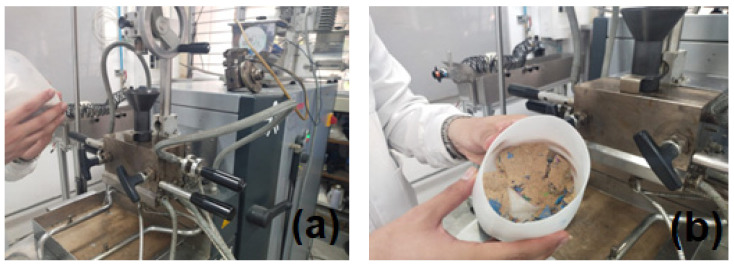
(**a**) Modular torque rheometer model Haake Poly 567-0016; (**b**) Example of WPC blend.

**Figure 2:**
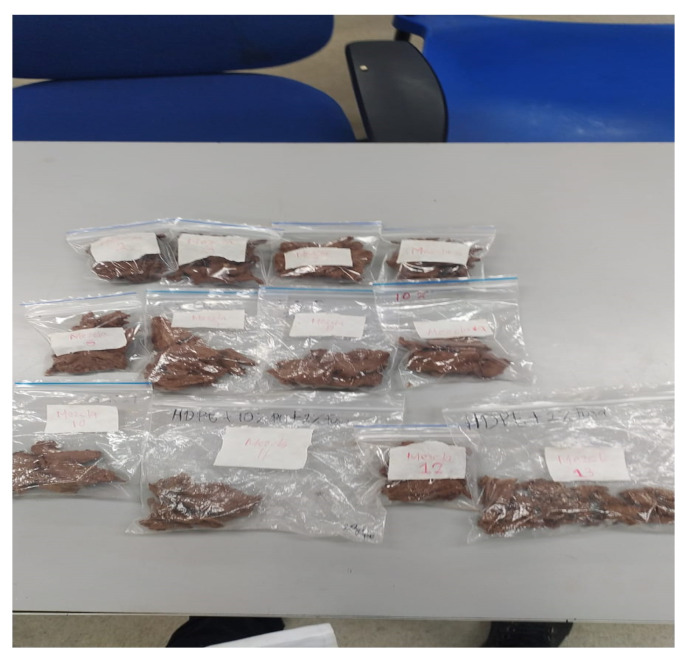
Blends from the modular torque rheometer.

The equipment used was a 77-kW modular twin-screw mixer (Modular Torque Rheometer System), model Haake Poly 567-0016 from the German brand Thermo Fisher (Waltham, MA, USA). The rheometer heating stage began while the components were pre-loaded into a vessel in the previously described order based on their specific surface area (SSA). Later, the vessel contents were emptied into the funnel of the rheometer’s inlet channel, previously heated to a temperature of 185 °C. Below in Figure 1 and Figure 2 the torque rheometer and the resulting mixtures are presented.

The rheometer parameters were 60 rpm and a residence time within the chamber of 5 min. Once the time elapsed, the mixture parts were extracted onto a stainless-steel tray and allowed to cool in the air for approximately 5 min. Subsequently, each mixture was stored in zip-lock bags.

The rheometer internal software allowed for the calculation of rheometric curves for each of the mixtures. Using the data provided by SENA, torque and temperature curves were plotted over time, as shown in the results analysis session of this document.

**Figure 3:**
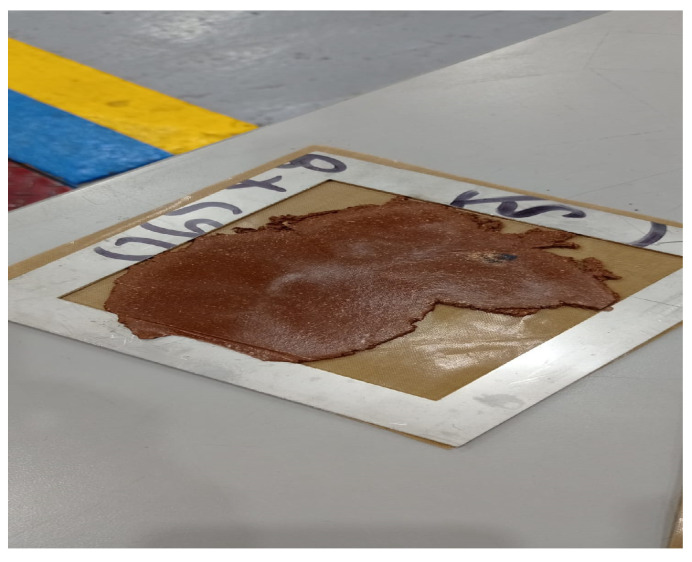
Plates after thermo-compression.

Finally, once the mixture was prepared, a thermocompression mechanism was employed. The material was placed between three metal plates: one compact upper plate, one compact lower plate, and one in the middle, shaped like the plate obtained after thermo-compression. Thin Teflon sheets were placed between each metal plate to prevent the material from sticking during the process. The temperature was raised to 185 °C to partially melt the material. The machine operator gradually reduced the temperature to control the pressure. Ultimately, the sheet was formed by thermocompression under well-controlled parameters. After approximately 5 min, the plate was allowed to cool without releasing the pressure for about 2 h, resulting in the final-shaped material as observed in the Figure 3.

In the stage following thermo-compression, the plates were machined using the same CNC machine as in the initial characterization stage. Between 3 and 5 test specimens were obtained to conduct the respective tensile tests, which served as the response variable for the experimental design.

## 3. Results and Discussion

Below we present Table 8 and Table 9, which compiles information on the properties of the initial formulation and reformulations 1 and 2 resulting from the experimental design (Section 3.2).

### 3.1. Characterization of the Initial Formulation

During this phase, the initial formulation was analyzed using physicochemical, thermal, and mechanical tests to determine the properties of the compound. Below are the test procedures and corresponding results.

#### 3.1.1. Physicochemical Analysis

##### Scanning Electron Microscopy (SEM)

To conduct this test, a sample was transversely fractured after being previously immersed in nitrogen. Subsequently, the fractured area was coated with gold to obtain the necessary results.

Through the images captured of the fracture zone in each sample, an adequate bond between the sawdust and the rHDPE matrix can be observed, thanks to the effective wetting derived from the low viscosity of the matrix. This low viscosity is a consequence of the reprocessing that occurs in the fusion of high-density polyethylene during the extrusion of the wooden plastic profiles because it has previously been melted during the blowing process to which it was initially subjected to form the containers. This reprocessing results in a loss of viscosity and therefore greater fluidity due to the shortening of the polymer chains due to thermal friction during the extrusion of the profiles. This facilitates adhesion between the materials even with high proportions of sawdust because the matrix can incorporate a high content of reinforcement within the composite material by completely wetting all the lignocellulosic material (sawdust). Coupling agents enhance the interaction between rHDPE (a hydrophobic polymer) and sawdust (a lignocellulosic material that is partially hydrophilic), resulting in better adhesion at the interface. Additionally, SEM analysis of the sawdust was performed to examine its geometry after grinding it for incorporation into the matrix. The results reveal that the crushed sawdust has elongated, needle-like shapes. Although this geometry suggests that the material could be acting as a reinforcement, it has not yet been determined whether its size actually contributes as a reinforcement or simply functions as a filler in the matrix.

**Figure 4:**
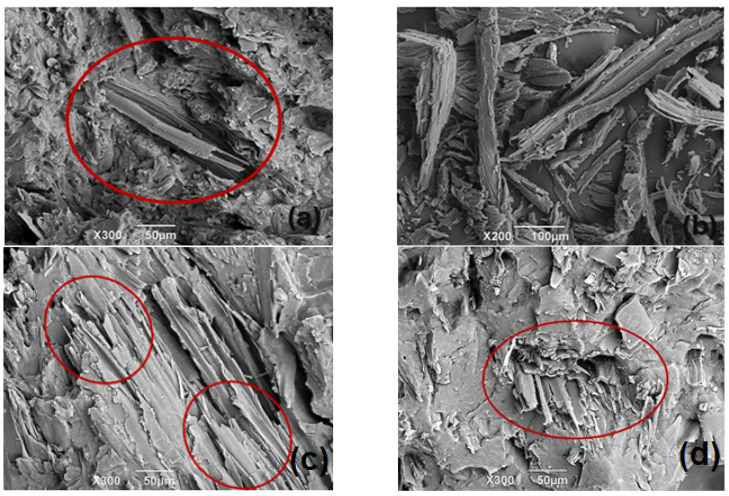
SEM microscopy of (**a**) initial rHDPE composite with sawdust fibers; (**b**) sawdust fibers; (**c**) formulation 1; (**d**) formulation 2. The red circle relates to the presence of sawdust fibers in the material.

Comparing the initial formulation with formulations 1 and 2 (Figure 4), it can be observed that in formulation 1 (Figure 4c), the fibers have greater wettability, indicating better interfacial interaction between the fibers and the matrix. In contrast, in formulation 2 (Figure 4d), cavities and gaps are observed between the fibers and the matrix.

Therefore, the results show that it is not necessary to use a large amount of coupling agent, such as maleic acid grafted, to achieve greater continuity between the composite materials. In formulation 1, 1.85% by weight fraction was used, while in formulation 2, 2.4% was used. This suggests that it is crucial to know the proportion ranges in which the composite can behave in different ways to achieve the best properties (such as water absorption, density, tensile strength, modulus of elasticity, Vicat, and HDT).

Thanks to the continuity between the fiber and the matrix, formulation 1 exhibits better properties compared with formulation 2 and the initial one.

##### Water Absorption

For composites that combine rHDPE and wood residues, it is crucial to understand their water absorption capacity. Moisture absorption refers to the material’s ability to capture water from the environment. In polymers, this moisture can function as a plasticizer, reducing the glass transition temperature and mechanical strength and even causing irreversible degradation of the structure.

In this study, type V specimens were selected, and the ASTM 570-22 [16] standard was used. Initially, the mass of the specimens was recorded using an analytical balance before immersing them in water for 24 h. After this period, the excess surface water was removed, and the samples were reweighed using the same balance. The environmental conditions in the laboratory during the experiment were as follows: ambient temperature 20–25 °C with relative humidity between 40% and 80%.

Since a substantial proportion of one of the composite materials, sawdust, is hygroscopic, the moisture absorption capacity was analyzed over a 24-h period. The results revealed a 2.032% moisture absorption after being submerged in water at this time. This finding suggests that combining a non-hygroscopic (hydrophobic) material in the matrix with a highly water-absorbent material leads to an increase in moisture absorption in the composite.

The results show that the hydrophilic hydroxyl group in wood sawdust absorbs water, causing swelling and an increase in the material’s dimensions and mass, thereby reducing its mechanical properties and limiting its use in humid environments. Moisture absorption increases over time until reaching a saturation point. The decrease in mechanical strength during prolonged exposure is more due to UV-induced bond rupture mechanisms than to the continuous increase of moisture within the composite material.

The pretreatment of natural fibers with chemical methods, such as using a silane coupling agent, improves adhesion at the fiber–matrix interface and reduces moisture absorption. This enhances the retention of the mechanical properties of the WPC. Additionally, moisture absorption can be significantly reduced by replacing a small amount of natural fibers with synthetic fibers.

In the study “Environmental Effects on Bamboo-glass/polypropylene hybrid composites” [25], it was found that hybrid WPC composites with a small amount of fiberglass reach a moisture saturation point after a certain period, maintaining constant moisture absorption from that point onwards. This phenomenon is also observed in wood plastic composites without synthetic reinforcements, although these have a higher percentage of moisture absorption due to their natural organic reinforcement.

According to the findings a composition that includes 24% HDPE, 70% sawdust flour, 4% maleic acid, and 2% other additives resulted in a moisture absorption of 0.05% in 24 h. However, in the initial formulation of this work, the moisture absorption increased to 2.032%. The highly water-absorbent behavior is a significant weakness of natural fiber composites, as these materials are susceptible to considerable water absorption due to their hydrophilic nature, leading to the degradation of mechanical properties related to strength and stiffness [26].

This suggests that a significant amount of wood flour can be used in the composite composition, but elevated levels of maleic acid are necessary to improve adhesion between the materials. By adjusting this formulation, the durability of the composite under humid conditions can be studied without compromising its properties, thanks to improvements in the composite’s morphology.

##### Density of the Composite Material

Density, a physical property, is related to the molecular structure of polymers. It refers to the amount of mass per unit volume, expressed in grams per cubic centimeter. To determine the true density of the composite material, the Anton Paar Ultrapyc 3000 helium pycnometer was used. This equipment employs the Archimedes principle of fluid displacement and Boyle’s law of gas expansion to accurately measure the volume and true density of the material. During the experiment, laboratory environmental conditions were maintained within the following ranges: temperature between 18 and 30 °C and relative humidity between 40 and 80%.

According to the results obtained, it was found that the density of the composite material belonging to the initial formulation was greater than the individual densities of the starting materials, with rHDPE of 0.96 g/cm^3^ and sawdust of 0.45 g/cm^3^, which results in a density of 1.29 g/cm^3^. The same behavior was observed for the density of formulations 1 and 2 obtained from the experimental design. This increase is attributed to a structural change towards a more compact shape, leading to a greater mass per unit volume and, consequently, an increase in the density of the material.

Since the initial materials are not compatible, it is necessary to incorporate coupling agents such as maleic acid-grafted PE. This allows for greater compatibility between the materials, resulting in higher density and lower porosity. This phenomenon is primarily due to improved dispersion and increased strength at the interfaces. Morphologically, it is observed that with lower amounts of coupling agent, there may be more porosity, resulting in a less compact material due to spacing between particles.

The density of a composite can vary depending on the proportion of coupling agent used, as noted by in their study [27]. They conducted various formulations of a composite based on HDPE and sawdust flour, finding density variations depending on the amount of HDPE and coupling agent used. For example, using 47% and 45% HDPE, 50% sawdust flour, and 3% and 5% MAPP, respectively, they obtained densities of 1.042 g/cm^3^ and 1.029 g/cm^3^.

Therefore, in the present work, using between 1.85 and 2.8% of coupling agent together with different proportions of HDPE and sawdust flour, a characteristic density of a non-porous and very compact material is achieved, which indicates good adhesion. between the initial materials. Below is the density obtained for the 3 composite materials studied (Table 10), and in comparison with two compounds with the same characteristics studied previously, it is observed that all have a density greater than 1 g/cm^3^ and less than 1.35 g/cm^3^.

##### Exposure of Samples to Weathering

The natural aging test was conducted following the guidelines established in ASTM D1435-20 [18], “Standard Practice for Outdoor Weathering of Plastics”. Test specimens were obtained by machining a pre-formed wood plastic profile according to dimensions recommended by the technical standard for tensile testing specimens.

**Figure 5:**
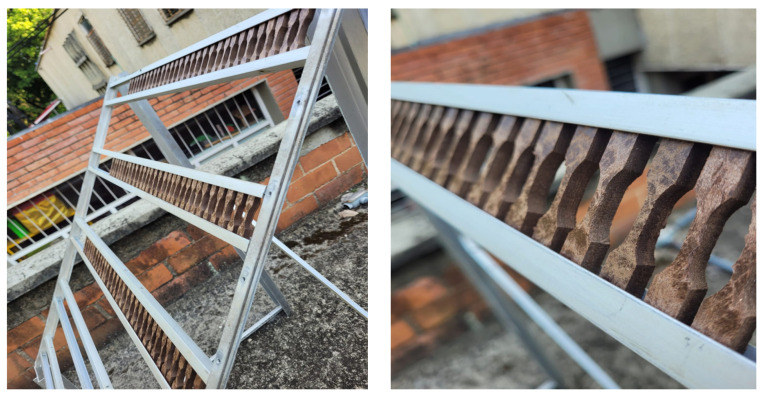
Exposure of samples for natural aging test.

A specific structure was built to house the specimens during the test, designed according to the guidelines of the natural aging standard for plastics. This structure was constructed using materials highly resistant to weather conditions, in this case, aluminum provided by the School of Materials Engineering at Universidad del Valle (Figure 5).

The structure was installed with maximum exposure inclination (45°), and the spacing between specimens was determined following the recommendations of the corresponding standard. It was placed on an unobstructed terrace exposed to UV rays, oriented towards the equator to ensure maximum exposure most of the time.

Procedure for Determining Mechanical Properties During Exposure:

- A tensile test under standard conditions, referred to as time-zero testing, was initially conducted to establish baseline strength.
- Five specimens were extracted from the aluminum structure in the first, third, fourth, fifth month, and subsequently the necessary months, and they were photographed for qualitative analysis in addition to performing the corresponding tensile tests.
- No control tests were conducted in the second month of exposure due to a break period in university activities, which made it difficult to coordinate activities due to a lack of personnel and supplies.
- Tensile tests of the specimens followed ASTM D 638-22 [22] at a speed of 5 mm/min on the universal testing machine in the mechanical testing laboratory of the School of Materials Engineering.
- After the tests, data related to the ultimate tensile strength and percentage elongation of the specimens were recorded for subsequent quantitative analysis.

Table 11 below shows the average tensile strength and maximum elongation for different months of exposure.

**Figure 6:**
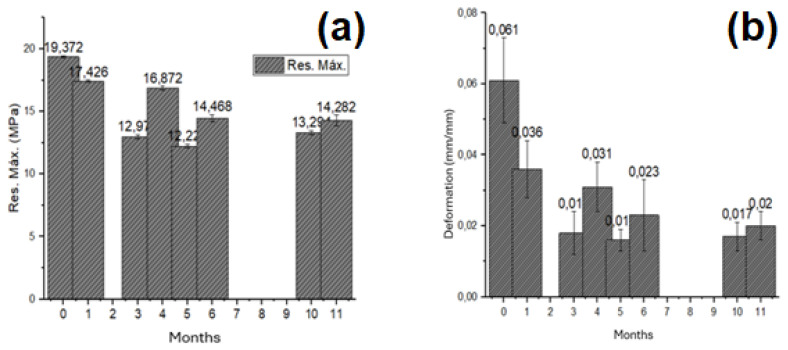
Variation of (**a**) stress and (**b**) strain as a function of time.

**Figure 7:**
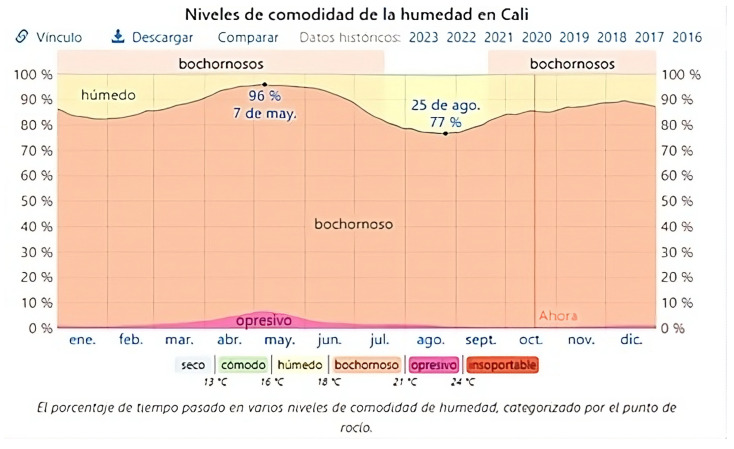
Relative humidity in the city of Cali in the year 2023 [28].

Figure 6 shows a decrease in maximum tensile strength and elongation for all months of exposure. However, an outlier was recorded in the fourth month, showing an increase compared with the previous month, although still lower than the first month of exposure. This outlier does not affect the general trend of decreasing mechanical properties. The following hypotheses have been proposed regarding this increase:

1. The relative humidity was lower during the fourth month of exposure (17 August to 17 September) compared with the previous month (17 July to 17 August), as shown in Figure 7. Lower relative humidity before testing can result in higher strength, as moisture ingress is reduced and the sawdust fibers are in better condition. However, this behavior is less likely over prolonged exposure periods, where long-term deterioration of the composite material predominates.
2. The increase in strength may be attributed to a greater preferential orientation of the fibers towards the direction of the applied stress in these specific samples. In other words, there was a higher proportion of fibers aligned parallel to the applied force, which increased the ultimate tensile strength.
3. The increase in strength compared with the previous month could be due to an operational error, resulting in this outlier in the research.

In “Environmental effects on bamboo-glass/polypropylene hybrid composites” [25], the effects of temperature and humidity on the thermal and mechanical properties of some polymeric composites reinforced with natural fibers are discussed. It has been shown that the degree of water sorption of NFRC (natural fiber-reinforced polymer matrix composites) depends on the relative humidity of the environment, the fiber volume fraction, the type of polymer used, the sorption time, and temperature. The mechanical properties (tensile, bending, and impact) of these compounds decrease restrictively with time and soaking temperature. The saturated moisture content and the level of deterioration are greater with aging at elevated temperatures. Pretreatment of natural fibers using chemical methods (e.g., use of a coupling agent such as a silane compound) improves adhesion at the fiber-matrix interface and reduces moisture absorption of these fibers. Consequently, the mechanical properties of NFRC under environmental aging are improved. Additionally, the amount of moisture absorption can be significantly reduced by replacing the natural fiber with a small amount of synthetic fiber, such as glass or carbon.

#### 3.1.2. Thermal Analysis

##### Vicat Softening Temperature

This test determines the temperature at which a 1 mm^2^ circular indenter penetrates 1 mm into the test sample under a standard load of 10 N or 50 N. To ensure the reproducibility of the data, four tests were performed using samples with dimensions of 7.80 mm thick, 20 mm long, and 8.90 mm wide in the case of the initial formulation. For formulations 1 and 2, specimens of the same dimensions but with a thickness of only 2 mm were used because these were obtained by thermocompression of plates that reached this maximum capacity, while the specimens for the initial formulation were extracted from a profile. The procedure followed the ASTM 1525-17 standard, using load 1 (10 N) or 1000 g and a heating rate of type B (120 °C/h) for high-density polyethylenes.

According to the aforementioned method, the samples recorded a penetration of 1 mm at temperatures of 129.4, 128.9, 128.8, and 129.7 °C, respectively, for the initial formulation. On average, a softening temperature of 129.2 °C was obtained. While for formulations 1 and 2, a softening temperature of 131.6 °C and 127 °C, respectively, was obtained.

The increase in the softening temperature of formulation 1 with respect to the initial formulation can be explained by the fact that in plate thermocompression there is a greater agglomeration of particles compared with the method of obtaining profiles by extrusion. This can increase hardness at high temperatures, more satisfactorily preventing the penetration of the penetrator. However, in the case of formulation 2, a decrease of a little more than 2 °C is evident, which can refute the previous hypothesis and explain that the increase and decrease in temperature is due to the sawdust content, mainly because formulation 2 is the one with the highest sawdust content of the 3 formulations studied.

In some cases, a moderate increase in sawdust concentration can cause an increase in the hardness of the WPC. This is because sawdust, being a rigid material, can help improve the resistance to deformation of the composite, thus increasing its hardness. On the other hand, some previous studies, such as “Physical and mechanical properties of polystyrene wood plastic composites -white oak wood flour” [29]. It suggests that there is an optimal concentration of sawdust to maximize hardness without compromising impact resistance too much. For example, a sawdust concentration in the range of 30–50% by weight may show improvement in hardness up to a certain point, after which hardness may begin to decrease. In the case of this study, it is established that even in the range of 50–65% of the sawdust weight fraction, the optimal hardness and softening temperature of the WPC studied are preserved.

This value aligns with the reported 129 °C for rHDPE in the Braskem Idesa technical data sheet. This result could be explained by two reasons. First, the area where the penetration occurred lacked a significant amount of sawdust, implying that only the rHDPE was affected. Second, the sawdust is affected at this temperature, meaning it does not contribute to resistance against Vicat needle penetration. Therefore, an increase in Vicat temperature would be expected if the sawdust distribution throughout the composite were uniform.

##### Heat Deflection Temperature (HDT)

This test can provide information on how polymeric materials behave under load at elevated temperatures. Using the dimensions of each sample, the equipment calculates the final desired deflection and records the temperature increase necessary to reach that value.

Including sawdust is anticipated to increase resistance to softening by temperature, similar to the effect observed with the Vicat temperature, as deflection occurs longitudinally where the load is distributed between the matrix and the reinforcement or filler. The results show a heat deflection temperature (HDT) of 125 °C under a load of 0.450 MPa with a deflection of 0.25 mm, compared with the 87 °C at 0.455 MPa reported in the rHDPE technical data sheet. This comparison suggests that the sawdust significantly contributed to the flexural resistance under temperature.

Therefore, in the previously analyzed Vicat temperature test, the results obtained seem consistent with the influence of sawdust, as indicated in the first analysis of the Vicat temperature.

When analyzing the results of Reformulations 1 and 2, differences can be observed because the initial formulation used a 6.63 mm thick sample, whereas the reformulations used samples approximately 2.36 mm thick. This changes the cross-sectional area during the test. In the reformulations, the conditions were 1.80 MPa with a deflection of 0.58 mm, obtaining the values reported in Table 9 (95.7 and 71.7 °C, respectively). Comparing the results of the reformulations with the initial formulation and considering the areas, Reformulation 1 stands out for having greater flexural stability when subjected to elevated temperatures.

As mentioned in the article by [30] titled “Manufacturing of wood plastic composite from polyethylene/poly (ethylene terephthalate) waste/ultra-high molecular weight polyethylene blends and rice husk fiber,” increasing the reinforcement content enhances the heat deflection temperature (HDT). There is a critical point where sufficient interfacial adhesion between the matrix and reinforcement is achieved. However, beyond this point, the HDT can decrease due to “fiber overload.”

In this study, similar results were anticipated. It was found that with a 55% reinforcement, the HDT was 71.7 °C, and increasing the content to 64.35% raised the HDT to 95.7 °C. Further testing is needed to determine the upper limit for fiber addition and to identify the deflection point in the HDT results.

##### Melt Flow Index (MFI)

This test is used to evaluate the flow capacity of a thermoplastic at a specific temperature. It provides information on the viscosity of the polymer at that temperature, which is linked to the molecular weight and, in turn, influences its properties. The test involves measuring the amount of material that flows through a cylinder over a given period, usually expressed in grams per 10 min (g/10 min).

For sample preparation, residues or cutting chips from the supplied profiles were used. The equipment used was a Genius Olsen extrusion plastometer, model MP600 with a microprocessor, operating at a temperature of 190 °C and a load of 21.6 kg, according to the ASTM D 1238-23 standard [21] (Table 12).

The inclusion of reinforcements in thermoplastics can affect the melt flow index, resulting in reduced material viscosity and, therefore, a decrease in the melt flow index (MFI). Therefore, in this composite, a reduction in the MFI is expected.

To observe these changes, the MFI was compared with a conventional HDPE material, and the HDPE was used as the matrix. The MFI of conventional HDPE was 6.2 g/10 min, while the matrix had an MFI of 32.12 g/10 min. This higher value is attributed to the fact that it is a recycled material, resulting in decreased properties due to thermomechanical effects and shortening of the polymer’s molecular chains. These results were obtained with a 21.6 kg load at a temperature of 190 °C.

Comparing these results with the composite, which showed an MFI of 3.26 g/10 min, confirms the initial hypothesis. The decrease in MFI indicates that the incorporation of sawdust increases the difficulty of processing this composite by reducing its viscosity, a crucial property in thermoplastic material processing.

The increase in processing difficulty due to the decrease in MFI is also manifested in the values obtained for formulations 1 and 2. In formulation 1, the fluidity index is lower than in formulation 2, as it has a 15% higher weight fraction of sawdust.

In addition to the advantages in terms of mechanical properties that are observed in formulation 1 with respect to formulation 2 and the initial one, it is shown that in terms of ease of processing, formulation 1 has advantages; it has an optimal fluidity index, which is in the appropriate range of fluidity expected for this type of compound despite having the lowest MFI of the three. This favorable condition is based on the fact that a higher melt index is the result of a low viscosity, which is often related to the deterioration of mechanical properties due to the low molecular weight of the polymer matrix. The above allows defining as a selection parameter for WPC formulations a condition in which the fluidity index belongs to a range of 3–4 g/10 min because, in the literature for WPC with HDPE matrix, the typical values of MFI are usually in the range of 1 to 5 g/10 min, with 1 to 3 being a limit of low fluidity and 4 to 5 a limit of high fluidity. Both limits can present difficulties in the extrusion process.

#### 3.1.3. Mechanical Analysis

##### Tensile Test

The principle of this test is to elongate a sample along its longitudinal axis at a constant rate until it breaks or fails. The maximum stress required is determined by the applied load and the deformation or elongation experienced by the sample.

For sample preparation, type V specimens were obtained from the supplied profiles, following ASTM 638-22 [22] guidelines. An H50KS universal testing machine was used, allowing the determination of the maximum stress a material can withstand before failing through the application of a load and the measurement of the longitudinal elongation of the piece. This destructive test was conducted with a 10 kN load cell and at a speed of 5 mm/min.

The tensile test results are presented in the section dedicated to sample exposure to weathering, specifically in month zero. This test is considered for two specific purposes: first, to evaluate the composite’s tensile strength; and second, to determine if the crushed sawdust acts as filler or reinforcement.

Commercial HDPE exhibits a reported strength of 31 MPa, while the composite shows a strength of 19.37 MPa. Initially, a notable decrease in tensile strength between the two materials is observed. However, since the composite contains HDPE recovered from a previous process, its mechanical properties are assumed to be intrinsically lower.

Furthermore, the incorporation of sawdust does not seem to significantly increase this property, suggesting that the sawdust’s contribution to the matrix should be analyzed due to findings by [31] in “Development and characterization of wood plastic composites with Pampas grass fibers” [31], which evidenced a progressive increase in both the modulus of elasticity and flexural modulus with an increase in natural fiber incorporation.

An 86% increase in the initial reported value of the modulus of elasticity for a 30% weight in fiber suggests that in addition to the chemical characteristics of the fiber and the incorporation of coupling agents, physical characteristics such as fiber length and pre-incorporation treatments could directly affect the mechanical performance of WPCs.

Kamal B Adhikary et al. in “Dimensional stability and mechanical behavior of wood plastic composites based on recycled and virgin high-density polyethylene (HDPE)” highlight that the tensile strength of wood plastic composites ranges between 9.5 and 23.2 MPa, with recycled high-density polyethylene composites exhibiting higher tensile strength than virgin high-density polyethylene composites without filler (23.2 and 21 MPa, respectively), possibly due to a higher proportion of rigid polyethylenes present in the recycled polyethylene [27].

The initial tensile strength without weathering exposure of 19.37 MPa is within the range reported in the literature. Additionally, the tensile strength of the composites increases as the wood content in the matrix decreases.

Similarly, another fundamental factor is the influence of incorporating a maleic anhydride coupling agent into wood plastic composites. This agent acts through an esterification process, forming ester bonds between the carbonyl groups in the coupling agent and the sawdust fibers. This reaction significantly improves the interfacial bonding between the matrix and reinforcement, increasing tensile strength and stiffness properties.

In the same previously indicated study, it was observed that composites with a weight range of 40–50% wood and MAPP exhibit the highest tensile strength and stiffness of all studied composites, being 60% greater in terms of strength than the same composite with the same range but without the use of MAPP.

The lower strength in composites without coupling agents is due to the lack of bonding between the reinforcement and the matrix, resulting in micro-defects that hinder the effective transfer of tensile stresses from the matrix to the fibers. The direct incidence of using coupling agents is a decrease in maximum deformation due to a significant increase in stiffness through an increased modulus of elasticity.

Finally, composites with higher HDPE content should typically exhibit greater ductility and maximum deformation because they have not been stiffened by the incorporation of wood flour or sawdust.

##### Impact Test

This dynamic test determines the resilience and toughness of the material, referring to its ability to withstand impacts and absorb energy before fracturing. It involves dropping a known mass pendulum, which strikes the sample placed on supports at the base of the equipment. Generally, materials that break into two halves are considered brittle, while those that bend without fracturing are considered ductile, meaning they have low brittleness.

For sample preparation, six specimens were made according to ASTM D256-10, with approximate dimensions of 8 mm in thickness, 11 mm in width, and 63 mm in length. These samples were not notched.

An IT504 Tinius Olsen pendulum impact machine was used following the unnotched Izod method. The equipment has a test speed of 3.46 m/s, a nominal energy range of 22.6 J at a height of 609.6 mm, and a nominal pendulum horizontal weight of 3770.5 g.

The average results for the composite show an impact resistance of approximately 34.64 J/m. While for the new formulations 1 and 2, the impact resistance per longitudinal unit was 16.35 J/m and 12.39 J/m, respectively, although the energy absorbed per unit area turned out to be higher for the new formulations, as can be observed in the following Table (Table 13).

For WPCs, typical Izod impact resistance values are typically in the range of 1 to 5 kJ/m^2^. The variability depends on the specific type of WPC and its properties. This result indicates that the inclusion of sawdust reduces both the material’s resistance and energy absorption capacity upon impact. This was also evidenced by [32] in a study on the mechanical properties of WPCs based on expanded polystyrene and sawdust or rice husk compared with other commercial WPCs and plywood. This study is detailed in the mechanical hardness testing section of the current study.

Vijaya observed that in terms of impact resistance, composites with a polymer matrix and sawdust or rice husk were significantly lower than plywood and other types of WPCs that do not use sawdust flour as reinforcement. Despite these WPCs being better at certain mechanical properties, such as hardness, the inclusion of sawdust reduces impact resistance.

The inclusion of sawdust can affect the impact resistance of the composite, especially considering that HDPE is known for its impact resistance, with typical values around 67 J/m. The distribution of sawdust throughout the matrix disrupts the formation of long, flexible molecular chains in the polymer, resulting in greater stiffness and a reduced ability to absorb energy.

Vijaya’s reported values show four fundamental facts regarding the impact resistance of these types of composites:

1. Plywood and typical commercial WPCs show an impact resistance of 74 and 52 J/m, respectively, while polystyrene and sawdust WPCs have a resistance between 40 and 52 J/m. This demonstrates that plywood is substantially better in terms of the amount of energy absorbed per unit length of material, while conventional composites have comparable impact resistance to the polystyrene matrix.
2. Composite material formulations with an expanded polystyrene matrix and a higher inclusion of sawdust perform worse in this mechanical property compared with the same material with a lower proportion of sawdust.
3. The impact resistance of the composite developed by Vijaya is approximately 27% higher than the composite material developed in the current research, indicating a possible satisfactory future application of polystyrene matrix WPCs over HDPE matrix WPCs.
4. None of the WPCs studied in this or other research articles achieve comparable impact resistance to plywood.

##### Hardness Test

Hardness refers to the ability of a material to resist penetration by other harder materials, that is, its resistance to surface deformations or breakage when point forces are applied.

For sample preparation and sampling, three samples were made following the guidelines of ASTM D2240 [23]. This test also aims to investigate how hardness is affected when the samples are submerged for 24 h.

A Shore durometer designed for polymers was used, with a 0.1 mm diameter sphere. Shore D hardness was measured by applying a 5 kg load, as it is a semi-rigid composite material. Multiple measurements were taken in different surface areas around each sample to obtain a representative average of the entire sample and not just a specific point.

The hardness of the composite can be positively or negatively influenced by the distribution of sawdust, as discussed in the Vicat temperature test. This test can serve as a complement to the one mentioned earlier. Compared with Braskem high-density polyethylene, which has a Shore D hardness of 65, the composite showed a value of 74 Shore D. This indicates that, depending on how the reinforcing material is distributed in the matrix, the hardness can notably increase. On the other hand, when the composite is submerged in water for 24 h, its hardness decreases to 72 Shore D. This could be due to the sawdust’s ability to absorb moisture, as mentioned in the water absorption analyses, resulting in a loss of composite properties.

Furthermore, Ref. [32] in “Compatibility studies of in-house prepared sustainable wood plastic composites with commercial composites” studied the hardness of a wood plastic composite based on expanded polystyrene and biomass from sawdust and rice husk and subsequently compared it with plywood and commercial WPCs.

They observed that the WPC developed based on high-density polyethylene and sawdust flour has a greater hardness than any of the seven types of compounds studied by Vijaya (2023). This is due to the higher intrinsic hardness of the matrix, as the hardness of HDPE alone is greater than that of polystyrene, which is specifically considered to produce plastic wood for characteristics other than hardness.

Additionally, Vijaya logically verified that a higher proportion of matrix within the composite provides greater hardness. This explains why, when comparing two WPCs with different matrices, the one composed of a material with higher hardness in the matrix will predominantly show higher hardness. For future studies, it is recommended to consider the ultimate purpose of the composite material regarding its application to determine the role of hardness and, therefore, the polymer matrix content in the manufacturing of the material.

### 3.2. Analysis of Results from the Reformulation of Plastic Wood

Following the initial characterization stage of the initial formulation, with the purpose of evaluating the effect of the formulation components of plastic wood on mechanical properties and moisture absorption, plates were made with the 13 formulations resulting from the experimental design, as observed in Table 7 of the experimental methodology section of this study. The preparation of the plates and the process parameters can be similarly observed in the previously mentioned section.

**Figure 8:**
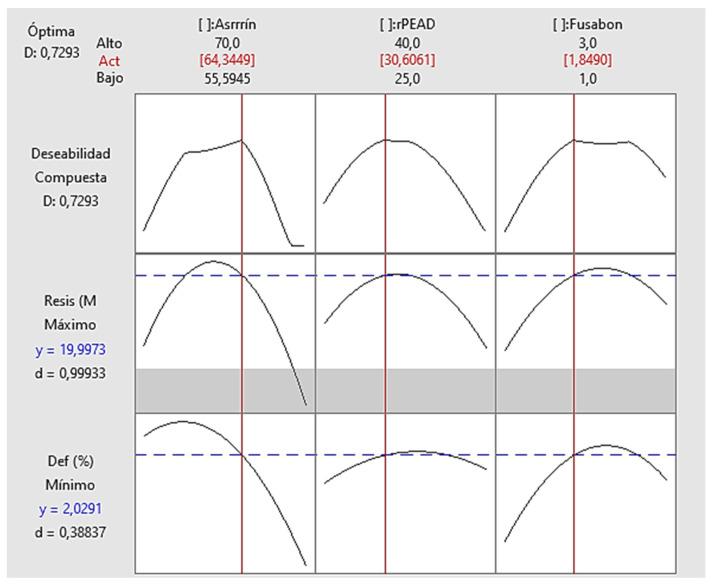
Tensile strength of each formulation.

The response variable of the experimental design was tensile strength, as shown in the following illustration (Figure 8).

With the results corresponding to the tensile strength and deformation of each formulation, the Minitab program was again used to obtain the two formulations with their corresponding compositions of each material in the WPC. This experimental design allows, through the scientific method, the obtaining of a specific composition of the composite material for property optimization. This allows for relating the material properties before and after the reformulation process. The two compositions optimized by the design were as follows (Table 14):

With a global and a local solution to the initial problem, plates were again prepared following the same procedure as described in the experimental methodology section. However, this time, it suffices to follow a comparative method involving both solutions, since the other possible formulations are discarded for not being part of either the global or local solution.

Subsequently, a tensile and hardness test was conducted as part of the mechanical analysis, and a 24-h moisture absorption test was conducted as part of the physicochemical analysis (Table 8).

## 4. Conclusions

The increase in tensile strength in some months during the natural aging test is due to the fact that in those samples, the fiber orientation was predominantly preferential towards the direction of the applied stress. That is, there was a higher fraction of fibers parallel to the stress, while in other specimens evaluated at a different time, the orientation was less preferential towards that direction, reducing the ultimate tensile strength. Nevertheless, the general behavior when exposed to the weather is a reduction in tensile strength due to UV degradation and repetitive moisture absorption processes.

The research and development of various formulations of wood plastic composite (WPC) materials, with variations mainly in the matrix and reinforcement, have led to the determination of an optimal formulation. Additionally, the incorporation of coupling agents, such as grafted PE, generates significant improvements as they allow for a better matrix-reinforcement interface. Therefore, through a systematic approach and the evaluation of various combinations, the ideal proportions that maximize mechanical properties have been identified.

Given that a substantial percentage of one of the materials (sawdust) in the composite is hygroscopic, the results show that when immersed in water for 24 h, there is a moisture absorption of 2.032% for the initial WPC formulation, while for the new improved formulations it was 1.8%.

Unlike what was later observed with the Heat Deflection Temperature (HDT) test, the final results of the softening temperature remain in a similar range because the resistance to penetration (hardness) is independent of the thickness of the material. However, it is observed that formulation 1 has a softening temperature of approximately 2.5 higher than the initial formulation. This is of great importance in allowing other properties to be improved, such as hardness at room temperature and moisture absorption, while maintaining the temperature and softening constant or even higher.

The sawdust content and its optimal concentration are critical factors in determining the hardness of wood plastic composites (WPCs). A moderate concentration of sawdust enhances hardness by strengthening the polymer matrix, whereas excessively high concentrations can have the opposite effect by compromising the material’s cohesion and its ability to resist deformation. For the present study, it is considered that the optimization range for sawdust content, typically between 20–50% by weight fraction, could be expanded. This is aimed at developing formulations for less demanding usage conditions, where mechanical properties are not essential, or to create formulations that, when combined with processes such as co-extrusion, result in products with acceptable mechanical properties, good weather resistance, and an increased incorporation of waste materials.

The results obtained so far serve as a guide for further evaluating the compound and achieving optimal properties according to its application. Depending on the application, its composition can be varied to obtain the desired response, as not all properties of the compound improve with an increased content of coupling agent or reinforcement. It is essential to understand the purpose of the material and the required response variables. Additionally, it is crucial to identify the upper and lower limits of the studied properties to determine the working range of the compound, considering a safety factor. In summary, these preliminary findings guide the adjustment of the compound’s composition to meet different applications, ensuring that key properties are optimized within a safe and effective range.

Finally, formulation 1 was considered with the weight proportion observed in Table 8 as the best possible formulation of plastic wood of the 14 formulations studied (13 yielded by the experimental design) and 1 corresponding to an initial or starting formulation. This formulation can be considered for future studies of plastic–wood composite materials with a matrix of high-density recycled polyethylene and sawdust or other similar WPCs as a starting basis when choosing the best proportion of materials to produce this type of material. Formulation 1 proved to be versatile both in processing and in such important properties as impact resistance, hardness, and water absorption, while retaining properties such as tensile strength, resistance to deformability, and penetration of heat.

## Figures and Tables

**Table 1:** Materials used in plastic materials.

Material	Characteristics
Recycled blown HDPE (rHDPE)	Being a material recovered from a post-industrial and post-consumer process, there is no specific reference. However, the company has a qualitative selection process where shredded pieces are chosen based on their color.
Sawdust	Like HDPE, sawdust is also recovered and comes from plywood. The sawdust is presented in shredded form.
Maleic acid-grafted PE	Fusabond P613, Dow Colombia
TPW Wax	TPW 813 coupling agent for plastic wood
Stearic Acid	Common stearic acid at 50% with 45% palmitic acid
Brown Pigment	Synthetic iron oxide brown pigment (α-Fe_2_O_3_/Fe_3_O_4_) in powder and micronized form

**Table 2:** Experimental methodology of physicochemical analysis.

Test	Experimental Methodology	Standard
Scanning Electron Microscopy (SEM)	The cross-section of a fractured sample of the composite material was observed using a JEOL JSM 6490LV scanning electron microscope. Samples were pre-coated with gold for better imaging since the material used is non-conductive.	Not applicable
Water Absorption	Sample conditioning was conducted in a water bath for 24 h at 50 °C, followed by cooling in a desiccator with silica gel and weighing on an analytical balance. After conditioning, specimens were fully immersed in distilled water at 23 °C for 24 h. After 24 h, they were dried superficially and re-weighed. The test ended when weight difference was <1% increase, or 5 mg.	ASTM D570 [16]
Density	This test was conducted using a helium pycnometer.	ASTM D5550 [17]
Weathering Exposure	Composite material samples were placed on frames of an aluminum structure inclined at 45 degrees. This structure was manually constructed by the researchers, and a monitoring and evaluation schedule for the samples was established over 3 months.	ASTM D1435 [18]

**Table 3:** Experimental methodology of thermal analysis.

Test	Experimental Methodology	Standard
HDT	The softening temperature HDT was determined using an HDT/VICAT TESTER model 1104 from Chengde Jinjian Testing Instrument Co., Ltd., Chengde, China. The samples were conditioned to dimensions of 80 × 12 × 2 mm and placed on the two supports for a 3-point bending of the sample.	ASTM D648 [19]
Vicat Softening Temperature (VST)	Vicat softening temperature was determined using an HDT/VICAT TESTER model 1104 from Chengde Jinjian Testing Instrument Co., Ltd., Chengde, China. Samples were conditioned to dimensions of 10 × 10 × 2 mm and placed in the holder with the indentation needle at the center of the sample.	ASTM D1525 [20]
Melt Flow Index (MFI)	Melt flow index test was performed using a Tinius Olsen MP600 Pantometer. A sample of 3.5 g was used with a test time of 1 min per sample.	ASTM D1238 (manual operation) [21]

**Table 4:** Experimental Methodology of Mechanical Analysis.

Test	Experimental Methodology	Standard
Tensile	Five specimens of the formulation were evaluated using a universal mechanical testing machine with a 10 kN load cell. Mean values of maximum strength, modulus of elasticity, and percentage elongation at maximum load were taken for experimental design analysis. Type IV specimens and a crosshead speed of 5 mm/min were used.	ASTM D638 [22]
Hardness	Hardness testing was performed on the Shore D scale using an HPSD-M model durometer from Electromatic Equip’t CO., Inc., Lynbrook, NY, USA. The durometer was pre-set on a stand to ensure the correct indentation height on the test specimens.	ASTM D2240 [23]
Impact	A halterio specimen was fixed at one end in a clamping device. A yoke was fixed at the other end. During the test, the pendulum hammer pulled the yoke, exerting a load on the specimen in tension until failure. Potential impact energy of the pendulum was used to move the yoke, and part of it was absorbed by specimen deformation.	ASTM D1822 [24]

**Table 5:** Composition limits in Minitab.

Component	Lower Limit (%)	Upper Limit (%)
Sawdust	55	70
rHDPE (recycled high-density polyethylene)	25	40
PE grafted with maleic acid	1	3
Wax (TPW)	0.9	0.9
Stearic acid	0.5	0.5
Pigment	1.8	1.8

**Table 6:** Initial formulation and runs with their corresponding composition % *w*/*w*.

Run	Sawdust	rHDPE	PE Grafted with Maleic Acid	Wax	Stearic Acid	Pigment
Initial	60.7	34.8	1.2	0.9	0.5	1.9
1	55.0	40.0	1.80	0.90	0.5	1.8
2	70.0	25.0	1.80	0.90	0.5	1.8
3	62.4	32.4	1.93	0.90	0.5	1.8
4	61.9	31.9	3.00	0.90	0.5	1.8
5	62.9	32.9	1.00	0.90	0.5	1.8
6	68.8	25.0	3.00	0.90	0.5	1.8
7	70.0	25.8	1.00	0.90	0.5	1.8
8	55.0	39.4	2.40	0.90	0.5	1.8
9	55.0	38.8	3.00	0.90	0.5	1.8
10	55.8	40.0	1.00	0.90	0.5	1.8
11	69.4	25.0	2.40	0.90	0.5	1.8
12	55.4	40.0	1.40	0.90	0.5	1.8
13	70.0	25.4	1.40	0.90	0.5	1.8

**Table 7:** Demonstration of calculated component ratios.

Blend 1	
Sawdust	14.73 g
rHDPE (recycled high-density polyethylene)	8.84 g
PE grafted with maleic acid	0.62 g
Wax (TPW)	0.23 g
Stearic acid	0.12 g
Pigment	0.45 g
Total	25 g

**Table 8:** Composition fraction by weight (%).

	Sawdust	rHDPE	PE Grafted with Maleic Acid. Wax	Wax	Stearic Acid	Pigment
Initial Formulation	60	34	2.8	0.9	0.5	1.8
Reformulation 1	64.35	30.6	1.85	0.9	0.5	1.8
Reformulation 2	55	39.4	2.4	0.9	0.5	1.8

**Table 9:** Properties of the initial formulation and reformulations 1 and 2.

	Absorption (%)	Density (g/cm³)	Tensile			Shore D Hardness	Impact Strength (KJ/m²)	Vicat (°C)	HDT (°C)
			Maximum Strength (MPa)	Strain at Maximum Load (mm/mm)	Elastic Modulus (MPa)				
Initial Formulation	2.03 ± 0.59	1.29	19.37 ± 1.68	0.06 ± 0.01	1427.50 ± 127.20	73.83 ± 1.47	4.30 ± 0.68	129.2	125.0
Reformulation 1	1.83 ± 0.47	1.20	15.73 ± 1.69	0.01 ± 0.01	2206.7 ± 304.60	85.00 ± 1.90	8.18 ± 2.25	131.6	95.7
Reformulation 2	2.41 ± 0.68	1.34	12.53 ± 1.88	0.01 ± 0.01	1782.1 ± 119.30	85.00 ± 4.00	6.46 ± 0.73	127.0	71.7

**Table 10:** Comparison of the densities of the WPCs studied.

Composite Material	Experimental Density (g/cm³)
Initial formulation	1.29
Reformulation 1	1.20
Reformulation 2	1.34
Similar composites in the literature	1.04

**Table 11:** Average tensile strength and maximum elongation for different months of exposure.

Month	Maximum Strength (MPa)	Strain at Maximum Load (mm/mm)	Elastic Modulus (MPa)
0	19.37 ± 1.68	0.06 ± 0.01	1427.50 ± 127.20
1	17.43 ± 1.42	0.04 ± 0.01	1116.04 ± 208.35
3	12.98 ± 2.29	0.02 ± 0.01	1348.10 ± 264.40
4	16.87 ± 2.55	0.03 ± 0.01	1086.07 ± 131.47
5	12.23 ± 1.96	0.02 ± 0.01	1313.60 ± 242.10
6	14.47 ± 3.85	0.02 ± 0.01	1183.60 ± 206.20
10	13.29 ± 2.35	0.02 ± 0.01	1321.90 ± 220.80
11	14.28 ± 6.27	0.02 ± 0.01	1402.10 ± 264.70

**Table 12:** MFI results.

Composite Material	Test Conditions	Result	Standard
Initial formulation	190 °C-21.6 kg	3.26 g/10 min	ASTM D1238-23 [21]
Formulation 1	190 °C-21.6 kg	3.105 g/10 min	ASTM D1238-23 [21]
Formulation 2	190 °C-21.6 kg	3.708 g/10 min	ASTM D1238-23 [21]

**Table 13:** Impact Strength Results.

Composite Material	Impact Strength (KJ/m²)
Initial formulation	4.30 ± 0.68
Reformulation 1	8.18 ± 2.25
Reformulation 2	6.46 ± 073

**Table 14:** Global and local design solutions.

**Global Solution**				**Local Solution**			
Sawdust			64.34	Sawdust			55.00
rHDPE			30.61	rHDPE			39.40
PE grafted with maleic acid			1.85	PE grafted with maleic acid			2.40
**Predicted Proposals**				**Predicted Proposals**			
Maximum Strength (MPa)	20.00	Desirability	1.00	Maximum Strength (MPa)	17.05	Desirability	0.40
Strain at Maximum Load (%)	2.03	Desirability	0.39	Strain at Maximum Load (%)	2.59	Desirability	0.17
Composite desirability	0.73			Composite desirability	0.30		

## Data Availability

All data are contained within the article.
